# *De novo *truncating mutations in *ASXL3 *are associated with a novel clinical phenotype with similarities to Bohring-Opitz syndrome

**DOI:** 10.1186/gm415

**Published:** 2013-02-05

**Authors:** Matthew N Bainbridge, Hao Hu, Donna M Muzny, Luciana Musante, James R Lupski, Brett H Graham, Wei Chen, Karen W Gripp, Kim Jenny, Thomas F Wienker, Yaping Yang, V Reid Sutton, Richard A Gibbs, H Hilger Ropers

**Affiliations:** 1 Human Genome Sequencing Center, Baylor College of Medicine, One Baylor Plaza, Houston, TX 77030, USA; 2 Department of Molecular and Human Genetics, Baylor College of Medicine, One Baylor Plaza, Houston, TX 77030, USA; 3 Max-Planck-Institute for Molecular Genetics, Ihnestraße, Berlin 14195, Germany; 4 Department of Pediatrics, Baylor College of Medicine, One Baylor Plaza, Houston, TX 77030, USA; 5 Texas Children's Hospital, 6621 Fannin, Houston, TX 77030, USA; 6 Max-Delbrueck-Centrum für Molekulare Medizin, Robert-Rössle-Straße, Berlin, 13092, Germany; 7 AI duPont Hospital for Children, 1600 Rockland Rd, Wilmington, DE 19803, USA

## Abstract

**Background:**

Molecular diagnostics can resolve locus heterogeneity underlying clinical phenotypes that may otherwise be co-assigned as a specific syndrome based on shared clinical features, and can associate phenotypically diverse diseases to a single locus through allelic affinity. Here we describe an apparently novel syndrome, likely caused by *de novo *truncating mutations in *ASXL3*, which shares characteristics with Bohring-Opitz syndrome, a disease associated with *de novo *truncating mutations in *ASXL1*.

**Methods:**

We used whole-genome and whole-exome sequencing to interrogate the genomes of four subjects with an undiagnosed syndrome.

**Results:**

Using genome-wide sequencing, we identified heterozygous, *de novo *truncating mutations in *ASXL3*, a transcriptional repressor related to *ASXL1*, in four unrelated probands. We found that these probands shared similar phenotypes, including severe feeding difficulties, failure to thrive, and neurologic abnormalities with significant developmental delay. Further, they showed less phenotypic overlap with patients who had *de novo *truncating mutations in *ASXL1*.

**Conclusion:**

We have identified truncating mutations in *ASXL3 *as the likely cause of a novel syndrome with phenotypic overlap with Bohring-Opitz syndrome.

## Background

**Figure 1:**
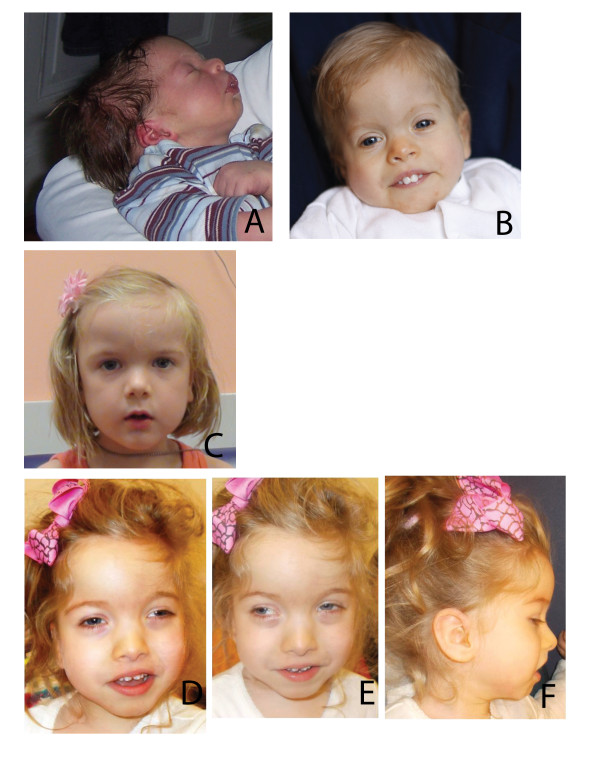
**Clinical presentation of four subjects**. **(A, B) **Subject 2 aged **(A) **1 month and (B) 38.5 months. Note the high forehead, low-set ears, thin arched eyebrows and anteverted nares. **(C) **Subject 3, aged 5 years, Note high and broad forehead, periorbital fullness, and anteverted nares. **(D-F) **Subject 4, aged 41.5 months, showing prominent tall forehead, arched eyebrows with subtle synophrys and periorbital fullness, prominent columella with hypoplastic alae nasi, thin upper lip, and borderline low-set ears. Note that none of the patients has trigonocephaly or prominent metopic ridge, as seen in Boehring-Opitz syndrome. Images were not available for subject 1.

Widespread use of high-throughput sequencing has helped elucidate the genetic heterogeneity underlying phenotypically similar syndromes. Bohring-Opitz syndrome (BOS; MIM 605039] is characterized by distinct craniofacial features and posture, severe intellectual disability, feeding problems, small size at birth, and failure to thrive [1], but shares some of these features with other syndromes. Recently, *de novo *truncating mutations in *ASXL1 *have been shown to account for approximatly 50% of cases with BOS [2];we initially and independently identified two individuals with *de novo *truncation mutations in a related gene, *ASXL3*. Subsequent interrogation of a small cohort identified two additional individuals with similar mutations. In all four families, the affected children had BOS-like features, but had no specific recognizable syndromic diagnosis. Subjects 1, 2, and 4 had similar clinical histories, including severe psychomotor retardation, feeding problems, severe post-natal growth retardation, arched eyebrows, anteverted nares, and ulnar deviation of the hands (Table 1, Figure 1), which are features partially shared with Cornelia de Lange Syndrome (CdLS) and BOS, but they did not have the trigonocephaly that is characteristic of BOS. Subject 3 also displayed anteverted nares, but had less severe psychomotor retardation and had normal growth. At 5 years of age, she has intellectual disability and does not speak. Magnetic resonance imaging data, which was only available for subject 4, showed cerebral volume loss over time, a feature seen in some cases of BOS (see Additional file 1, Figure S1). In the exome data of any of the subjects, no rare variants were found in the genes known to cause BOS and CdLS.

**Table 1:** Parental age at conception and gestation, and phenotypes of affected subjects and of typical BOS.

	Subject 1	Subject 2	Subject 3	Subject 4	BOS
Parental age, years	45/44	30/29	24/29	26/26	
Gestation	38 2/7 weeks; poor fetal growth; polyhydramnios	39 weeks, breech birth	38 weeks, C-section	40 weeks, spontaneous vaginal delivery	
Size at birth	*~1%*	*~1%*	40%	Weight 50%; length 25 to 50%	*Approximately 1%*
Gastrointestinal	*Gastric tube feeds for 8 months, gastro-esophageal reflux *	*Gastric tube feeds first month*	No obvious difficulties	Difficulty latching on from birth; poor oral feeding resulted in admission at age 8 weeks for failure to thrive; *gastro-esophageal reflux*; Nissen fundoplication and *G-tube *placed	*Feeding difficulties*
Craniofacial features	*Arched, thin eyebrows; high, narrow palate; low, posteriorly rotated ears; microcephaly; anteverted nares*, large fontanels; retroganthia; long eyelashes	*Arched eyebrows; high, narrow palate; low, posteriorly rotated ears; microcephaly; anteverted nares; *hypertelorism; *short nose*; gingival hyperplasia; sparse hair; scaly scalp; hyperopia	Prominent forehead/frontal bossing; *short nose *with *anteverted nares*	*Prominent forehead; arched eyebrows; *hypoplastic alae nasi; *low-set ears; high and narrow palate*	*High, narrow palate; low-set, posteriorly rotated ears; microcephaly; *trignocephaly; *anteverted nares*; prominent eyes; upslanting palpebral fissures; depressed nasal bridge
Somatic features	*Ulnar deviation of hands at rest; deep palmar *and plantar creases; hypertonia; bladder dysfunction; testes normally descended	*Ulnar deviation of hands; deep palmar creases; hypotonia*; *clenched hands; *undescended testes	Mild global *hypotonia*	Exotropia; *hirsutism; *supranumerary nipple; low truncal tone with abnormally fluctuating tone in limbs; no clonus; *mild ulnar deviation of the hands*; *deep palmar creases and clenching of hands*, less prominent over time	High myopia; *hirsutism*; 'BOS' posture: exorotation and/or adduction of the shoulders; flexion at the elbows; flexion at the wrists; and *ulnar deviation of the hands* and/or fingers at the MCP joints
Post-natal growth & development	*Weight, length & OFC all -4.5 to -5.0 SD; psychomotor delay*. Died aged 9 months.	*Size < 1%;^a ^severe psychomotor delay*	*Normal growth; global developmental delay with intellectual disability*	Age 41.5 months: *OFC < 2^nd^%; *50^th ^% for age 1 year; Length, weight 10 to 25 th percentile; unable to sit independently; non-verbal; no sign language; mostly G-tube fed	*Growth retardation*^a^; *psychomotor delay; high rate of infant mortality*
Brain imaging results				*Global mild white-matter volume loss *with normal myelination; secondary brainstem hypoplasia; hypoplasia/dysplasia of bilateral cerebellar tonsils; mild inferior vermian hypoplasia; normal MR spectroscopy	Enlarged ventricles, agenesis of corpus callosum, Dandy-Walker malformation, delayed myelination, and *cortical atrophy*
Laboratory testing with non-diagnostic results	*NIPBL *sequencing (Cornelia de Lange)	aCGH; urine oligosaccharide; sequencing panel for Noonan, Prader-Willi, cardiofaciocutaneous, and Costello syndromes; thin-layer chromatography for fucosidosis, mannosidosis, aspartylglucosaminuria, GM1- and GM2-gangliosidosis, galactosialidosis, Schindler and Pompe disease; [AU What does 'M' stand for here? Are these Schindler disease and Pompe disease? Ms have been removed ] electrophoresis for congenital disorders of glycosylation	SNP aCGH; methylation for Angelman; plasma and urine creatine and guanidinoacetate; urine purine and polyol panels; plasma homocysteine	46, XX; SNP aCGH; Sequencing panel for congenital disorders of glycosylation; N-glycans; methylation for Angelman syndrome; MECP2 sequencing; muscle biopsy gave normal electron and light microscopy results; urinary organic acids; plasma amino acids; CPK	

aCGH, array-comparative genomic hybridizationl BOS, Boehring-Opitz; CPK, creatine phosphokinase; G-tube; gastric tube; MCP, metacarpophalangeal; MECP, methyl CpG binding protein; MR, magnetic resonance; OFC, occipitofrontal circumference; SD, standard deviation; SNP, single-nucleotide polymorphism.

Features shared by at least two subjects and BOS are in italics.

## Methods

### Ethics approval

The study was approved by the Baylor Institutional Review Board (IRB), by the appropriate ethical committee at the MPIMG, and informed consent was obtained from the guardians of all subjects.

### DNA

DNA from subjects and their parents was obtained under written informed consent, provided by their parents or legal guardians, for participation in the study. The parents or legal guardians consented to publication of their children's images and clinical details. The study was approved by the Institutional Review Board at Baylor College of Medicine for all sequencing conducted at the Baylor College of Medicine Human Genome Sequencing Center (BCM-HGSC). This study was conducted in accordance with the Helsinki declaration.

### Library construction

Library construction was carried out at BCM-HGSC. After determining DNA concentration and integrity, high molecular weight double-stranded genomic DNA samples were constructed into pre-capture libraries (PairEnd; Illumina Inc., San Diego, CA, USA) in accordance with the manufacturer's protocol with some modification. Briefly, 1 μg genomic DNA in a volume of 100 μl was sheared into fragments of approximately 300 bp in a 96-well plate with an automatic processor(E210 system; Covaris, Inc. Woburn, MA, USA), using the 10% duty cycle setting, with an intensity of 4, and 200 cycles/burst for 120 seconds. Fragment size was evaluated using a 2.2% gel cassette (FlashGel DNA Cassette; catalog No. 57023; Lonza Group, Boston, MA, USA). The fragmented DNA was end-repaired in 90 μl total reaction volume containing sheared DNA, 9 μl 10× buffer, 5 μl enzyme mix (END Repair Enzyme Mix; New England Biolabs, Beverly, MA, USA) and H_2_O (NEBNext End Repair Module; catalog no. E6050L; New England Biolabs) and then incubated at 20°C for 30 minutes. A-tailing was performed in a total reaction volume of 60 μl containing end-repaired DNA, 6 μl 10× buffer, 3 μl Klenow Fragment (NEBNext dA-Tailing Module; catalog no. E6053L; New England Biolabs), and H_2_O, followed by incubation at 37°C for 30 minutes. Illumina multiplex adapter ligation (NEBNext Quick Ligation Module catalog no. E6056L;; New England Biolabs) was performed in a total reaction volume of 90 μl containing 18 μl 5× buffer, 5 μl ligase, 0.5 μl 100 μmol/l adaptor, and H_2_O at room temperature for 30 minutes. After ligation, PCR with primer PE 1.0(Illumina) and modified barcode primers (manuscript in preparation) was performed in 170 μl reactions containing 85 2× PCR master mix (Phusion High-Fidelity; New England Biolabs), adaptor-ligated DNA, 1.75 μl of 50 μM each primer and H_2_O. The standard thermocycling for PCR was 5 minutes at 95°C for the initial denaturation followed by 6 to 10 cycles of 15 seconds at 95°C, 15 seconds at 60°C and 30 seconds at 72°C, with a final extension for 5 minutes at 72°C. Magnetic beads (Agencourt^®^ XP^®^ Beads; catalog no. A63882; Beckman Coulter Genomics, Inc., Danvers, MA, USA) were used to purify DNA after each enzymatic reaction. After bead purification, PCR product quantification and size distribution was determined using a DNA assay (GX 1K/12K/High Sensitivity Assay Labchip; catalog no. 760517; Caliper, Hopkinton, MA, USA).

### Exon capture

At BCM, 1 μg of Illumina paired-end pre-capture library DNA was hybridized to a custom capture reagent designed at the HGSC and constructed by Roche/NimbleGen (reference no. 9999042355 Madison, WI, USA). This reagent targets the coding regions of genes reported by CCDS, ENSEMBL, UCSC, GenCode, VEGA, and RefSeq. It also targets micro RNA (miRNA) and small nucleolar RNA detailed by UCSC, predicted miRNA binding sites, and 1000 Sanger-predicted miRNAs. This design is available upon request. Hybridization was conducted in accordance with the manufacturer's protocol, (NimbleGen, Madison, WI, USA) with minor revisions. Specifically, three hybridization-enhancing oligos, IHE1, IHE2, and IHE3, (manuscript in preparation) replaced oligos HE1.1 and HE2.1, and post-capture LM-PCR was performed using 14 cycles. Capture libraries were quantified (GX 1K/12K/High Sensitivity Assay Labchip; catalog no. 760517; Caliper). The efficiency of the capture was evaluated by performing a quantitative (q)PCR-based quality check on the built-in controls (qPCR SYBR Green assays; Applied Biosystems, Foster City, MA, USA). Four standardized oligo sets, RUNX2, PRKG1, SMG1, and NLK, were used as internal quality controls. The enrichment of the capture libraries was estimated to range from seven-fold to nine-fold over background.

### Whole-exome sequencing

Whole-exome sequencing was carried out at the Max Planck Institut für molekulare Genetik (MPIMG). Whole-exome sequencing libraries were prepared (SureSelect XT Target Enrichment System for Illumina Paired-End Sequencing Library; Agilent Technologies Inc., Wilmington, DE, USA). Genomic DNA samples were used to generate Illumina PairEnd pre-capture libraries in accordance with the manufacturer's protocol (Agilent Technologies). After determining concentration and quality, 3 μg genomic DNA was sheared into fragments with an average length of 150 and 200 bp using a Covaris S2 system (Covaris, Inc. Woburn, MA). The setting was 10% duty cycle, with an intensity of 5, and 200 cycles/burst, for 360 seconds. After purification using magnetic beads (Agencourt AMPure XP; catalog No. A63882; Beckman Coulter Genomics), fragment size was checked using a bioanalyzer (2100; Agilent). The fragmented DNA was end-repaired in 100 μl total reaction volume containing 48 μl sheared DNA, 10 μl 10× buffer, 1.6 μl dNTP, 1 μl T4 DNA polymerase, 2 μl Klenow DNA polymerase and 2.2 μl T4 polynucleotide kinase, and then incubated at 20°C for 30 minutes. A-tailing was performed in a total reaction volume of 50 μl containing end-repaired DNA, 5 μl 10× buffer, 3 μl Klenow fragment, 1 μl dATP, and 11 μl water, followed by an incubation at 37°C for 30 minutes. Illumina adapter ligation was performed in a total reaction volume of 50 μl containing 10ul 5× buffer, 10 μl ligase and 10 μl adaptor oligo mix at room temperature for 30 minutes. After ligation, PCR with Illumina PE 1.0 and reverse primer (SureSelect GA Indexing Pre Capture PCR Reverse Primer; Agilent Technologies) was performed in 50 μl reactions containing 10 μl 5× buffer (Herculase II Rxn; Agilent Technologies), adaptor-ligated DNA, 1.25 μl of each primer, 0.5 100 mmol/l dNTP mix and 1 μl DNA polymerase (Herculase II Fusion; Agilent Technologies. The standard thermocycling for PCR was 2 minutes at 98°C for the initial denaturation, followed by six cycles of 30 seconds at 98°C, 30 seconds at 65°C, and 60 seconds at 72°C, with a final extension for 10 minutes at 72°C. Magnetic beads (Agencourt^®^ XP^®^ Beads; Beckman Coulter Genomics; catalog no. A63882) was used to purify DNA after each enzymatic reaction. After bead purification, PCR product quantification and size distribution was determined using a bioanalyzer (model 2100; Agilent Technologies) and Qubit software (University of Oxford, Oxford, UK), then 500 ng of the Illumina paired-end pre-capture library DNA was hybridized to Agilent SureSelect human exome capture probes in accordance with the manufacturer's specifications. After assessing the quality of capture libraries using a bioanalyzer (2100; Agilent Technologies), the captured library was sequenced using a sequencing system (Hi-Seq 2000; Illumina) in accordance with the manufacturer's protocol. Each library was sequenced in a separate lane in a 101-nucleotide single-end sequencing format.

The whole human genomes were resequenced by a commercial company (Complete Genomics, Mountain View, CA, USA) with unchained base reads on self-assembling DNA nanoarrays [3], and the variants called by using mated gapped reads [4].

### Sequence alignment, variant calling, annotation, and verification

At BCM, Illumina data was aligned by use of Burrows-Wheeler Aligner (BWA) software. Variants were called using ATLAS-SNP v2.0 and the SAMtools program Pileup. *De novo *variants were found by *in silico *subtraction of the variants discovered in either parent. Variants were subsequently annotated for effect on the protein, known minor allele frequencies, and gene function using AnnoVar and custom in-house developed software. Candidate variants were verified and segregation examined using Sanger capillary sequencing.

At MPIMG, the exonic region of human genome was enriched (SureSelect Human All Exon Kits; Agilent Technologies), and deep sequenced (HiSeq 2000; Illumina). Raw sequence reads were pre-screened to remove low-quality reads, and then aligned to the human reference genome with SOAP (version 2.21). Aligned and unaligned reads were used to call the single-nucleotide variants (SNVs) and Indels, respectively. Variant lists were filtered against the reference databases and ranked as potential candidates [5].

The primary variant list from Complete Genomics was filtered and prioritized by an in-house pipeline [5]. *De novo *mutations in each patient were defined as those absent from all other members in the family and that had been flagged as 'high quality' and supported by at least five reads with allele percentage of 0.4 to 0.6. Candidate variants were verified and segregation tested using Sanger capillary sequencing.

### RNA extraction and reverse transcriptase-PCR experiments

A cell line from subject 2 was established by Epstein-Barr virus transformation, in accordance with standard protocols after informed consent. Total RNA was extracted from patient cells using a commercial kit (RNeasy Plus; Qiagen Inc., Valencia, CA, USA) in accordance with the manufacturer's recommendations. For reverse transcription, 1 μg of RNA was used with 10 U of Superscript III (Invitrogen Corp., Carlsbad, CA, USA), and 150 μmol/l random hexamers in the presence of ribonuclease inhibitor (RNasin; Promega Corp., Madison, WI, USA) in accordance with the manufacturer's protocol. Reverse transcriptase (RT)-PCRs for allele expression analysis of ASXL3 were carried out using a PCR mix (BIO-X-ACT Long Mix; Bioline Reagent Ltd, London, UK) and primers (ASXL3_RTmut_for 5'-AAATGCAGTTGCGGATAAGG-3' and ASXL3_RTmut_rev 5'-TGGGGTTCTTCATGAGAATTC-3'), located in exons 10 and 11. After initial denaturation at 94°C for 3 min, cycling conditions were: 40 cycles at 94°C, 55°C and 72°C. Each step was for 45 seconds.

### *ASXL3 *analysis

Exon data for the gene and prosite regions was extracted from the ENSEMBL genome browser [6]. Vertebrate conservation was obtained from the UCSC Genome browser [7], using the vertebrate conserved elements track (Vertebrate Multiz Alignment & Conservation (46 Species): Vertebrate Conserved Elements) with a minimum LOD score of 700. Predicted motifs were obtained from the eukaryotic linear motif [8] server with a motif probability cut-off of 0.005. All phosphorylation events were grouped together. ASXL family similarity was calculated by BLASTing ASXL1 and ASXL2 amino acid sequences against ASXL3 using NCBI-BLASTP[9].

## Results and discussion

To identify potential causative alleles, genome sequencing was undertaken in each family we identified (see Additional file 2, Table S1). For family 1, we interrogated the exome of both parents and the affected child using a custom exon-capture reagent, followed by high-throughput sequencing with the Illumina HiSeq platform. For family 2, we obtained the whole genome sequence of the affected child, his unaffected sibling, and both parents from Complete Genomics. We also performed exome sequencing in this family as part of a pilot study to compare the yield of these methods (see Methods). For families 3 and 4, exome sequencing was performed on the proband only. After sequencing, we identified all coding and near-intronic differences between the subjects and the human reference genome (see Methods). By using the unaffected parents as controls, it was possible to identify *de novo *mutations in the affected subject.

In each generation, 70 to 175 *de novo *point mutations are expected [10,11], and 0 to 3 of these are anticipated to cause protein-coding changes [11]. In subject 1, a single protein-coding *de novo *mutation was identified (chr18, g.31318578C > T, p.Q404X; hg19). In subject 2, two coding *de novo *mutations were identified. The first (chr16, g.75258715G > A, p.R248H) occurred in *CTRB1*, and was considered non-pathogenic, as the variant has been reported in samples analyzed for the Thousand Genomes project (rs191950160), and is identical to the orthologous alleles in the chimpanzee and rhesus macaque. The second occurred in *ASXL3 *(chr18, g.31318764C > T, p.Q466X) and has not been previously reported. In subject 3, a *de novo *4 bp deletion was identified (chr18, g.31319343_31319346delACAG, p.T659fsX41); this frameshift was predicted to generate a premature termination codon (TGA) after an additional 41 amino acids. In subject 4, a *de novo *1 bp insertion was identified (chr18, g. 31318789_insT, p.P474fs); this frameshift mutation was predicted to generate a premature termination codon immediately. In all cases, the variant was interrogated in the affected child and parental DNA obtained from peripheral blood by Sanger capillary sequencing. All four *de novo ASXL3 *mutations generated stop codons, and were predicted, *in silico*, to generate a truncated ASXL3. Further, the mRNAs containing these premature stop-codon mutations may be degraded by nonsense-mediated decay (NMD) and thus could represent loss-of-function alleles. Neither SNV allele occurred in a CpG dinucleotide; the only known site with an increased propensity for *de novo *mutations [10] (see Additional file 3, Table S2).

In *Drosophila*, the additional sex combs (*Asx*) gene is required to maintain homeotic gene activation and silencing, and in mice, three orthologs (*Asxl1, Asxl2*, and *Axl3*) have been identified. Asxl1 acts on the developmentally important *Hox *genes both as a repressor (*HoxA4, HoxA7*, and *HoxC8*) and as an enhancer (*HoxC8*) [12]; dysregulation of the human *HOX *genes may account for the developmental phenotype. Little information is available about either embryologic or fetal expression of the *ASXL *gene family; however, in *Drosophila*, regulation of the *Asx *gene is highly variable and tightly controlled in the first 3 hours after fertilization [13]. In humans, ASXL3, like ASXL1 and ASXL2, is a putative polycomb protein and probably acts as a histone methyltransferase in a complex with other proteins [14]. *ASXL3 *is expressed in similar tissues to *ASXL1 *including brain, spinal cord, kidney, liver, and bone marrow, but at a lower level [15] (see Additional file 4, Figure S2). Within the brain, *ASXL1 *has much higher expression in the white matter, whereas *ASXL3 *has moderately higher expression in the insula, cingulate gyrus, and amygdala, with approximately similar expression elsewhere [16] (see Additional file 5, Figure S3). The high correlation of expression patterns between *ASXL1 *and *ASXL3 *may account for some of the shared phenotypic features.

**Figure 2:**
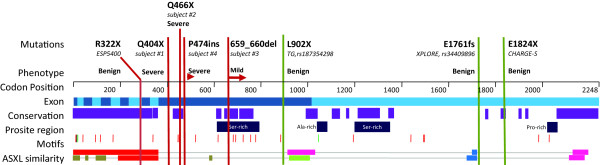
**Known nonsense mutations in *ASXL3***. Known nonsense mutations are shown (dotted line) across the *ASXL3 *gene, and are given with the phenotypic effect and DNA source. Amino acid position is listed (top) with exon position (light/dark blue). Regions of high vertebrate conservation are shown (purple) along with Prosite regions (dark blue), and conserved and predicted motifs (nuclear localization in black, ASXL-specific motifs in green, phosphorylation sites in red) amino acid similarity between ASXL3 and ASXL1 (top) and ASXL2 (bottom) (highest similarity to lowest: red, pink, green, blue, brown). Adapted from UCSC Genome browser, ENSEMBL, eukaryotic linear motif server, and NCBI-BLASTP.

No deleterious *ASXL3 *mutation was found in a small cohort of patients with BOS without causative *ASXL1 *mutation (Hoischen, personal communication), consistent with *ASXL3 *mutations conveying a phenotype distinct from BOS. Using large-scale datasets (Thousand Genomes [17], dbSNP, ESP5400, and Cohorts for Heart and Aging Research in Genomic Epidemiology [18]) we identified four other truncating mutations in *ASXL3*, which occurred as singletons within each dataset in reportedly phenotypically normal individuals (Figure 2), and thus may represent benign variants. Two of these mutations occur at the extreme 3' of the gene, and thus may escape NMD and retain protein activity. One mutation, p.L902X (rs187354298), identified in a single sample from the Thousand Genomes cohort, occurs more 3' to, but within the same penultimate exon as, the two disease-causing mutations (see Figure 2). More interestingly, however, a high-quality nonsense mutation (R322X) was identified in the exon 9 of the 12-exon *ASXL3 *gene, and is anticipated to undergo NMD or may be otherwise highly deleterious to protein function.

Although all four disease-associated *de novo *variants and rs187354298 are predicted to potentially undergo NMD, it is challenging to reconcile such a hypothesis with the observed range of phenotypes for nonsense alleles reported at this locus. However, the current ability to predict NMD is limited, and it has been shown that around 75% of mRNA transcripts that are predicted to undergo NMD escape destruction, and that the nonsense codon-harboring mRNA is expressed at levels similar to wild type in lymphoblastoid cells [19]. Furthermore, the dynamics of mRNA stability and degradation may differ for cells and tissues undergoing rapid developmental changes. Parenthetically, we noted an enrichment of around 50% in gene regions where mRNA would be predicted to escape NMD, or 3' gene bias to the predicted loss-of-function nonsense codon mutations in normal controls from the Thousand Genomes data [19].

Another distinct interpretation of our observations is that all *ASXL3 *disease-causing nonsense-encoding mRNAs are translated into prematurely terminated proteins, which act in a dominant-negative fashion. In support of this hypothesis, Sanger sequencing of multiple cDNA extractions derived from a transformed lymphoblast cell line from subject 2 showed that both alleles were expressed (see Additional file 6, Figure S4) although this does not exclude that some degree of NMD may occur or reflect what occurred during development. Further, we observed that in previously reported cases of BOS known disease-causing nonsense mutations in *ASXL1 *[2,20] occur almost entirely within a very limited region of the protein,. Furthermore, database searches reveal that truncating mutations, in reportedly phenotypically normal individuals, can occur both 5' and 3' of these mutations, just as we now report for *ASXL3 *(see Additional file 7, Figure S5). This disease-causing mutation hotspot falls between two paralogous regions shared by all *ASXL *genes (Figure 2) and into a region unique to *ASXL1*. Interestingly, the presumptive disease-causing mutations we describe here occur within an analogous region in *ASXL3*, within the first half of the penultimate exon. Further, disease severity may decrease the more 3' the mutation occurs within this region. This region contains a number of predicted phosphorylation sites, an evolutionarily conserved region (residues 420 to 470, approximately), and an evolutionarily conserved serine-rich motif between residues 600 and 800, approximately. We speculate that disruption of these conserved regions may result in dysregulation of post-translational protein modification, resulting in constitutive activation.

Truncating *ASXL3 *mutations are uncommon, and their *de novo *nature makes it even less likely that we identified these individuals by chance, which highlights the value of *de novo *mutation-based methods to find disease-causing loci. To determine the probability of observing multiple *de novo *truncating mutations in *ASXL3*, we developed a model [21,22] accounting for gene size, GC content and *de novo *rates of SNVs and small insertion/deletions, and of the probability of those mutations causing a truncation of the protein. The probability of developing a *de novo *nonsense mutation in *ASXL3 *is 3.35 × 10^-6 ^per generation, whereas the probability of developing a *de novo *coding insertion or deletion in *ASXL3 *is approximately 3.91 × 10^-6^. Thus, the total probability of observing three additional individuals with truncating *ASXL3 *mutation, given the first *de novo *observation, is around 4.0 × 10^-17^. The observation of four *de novo *truncating mutations occurring in association with a sporadic disease that shares similar phenotypic features is highly unlikely to have occurred by chance; nevertheless, functional studies will be required to show conclusively that truncating mutations in ASXL3 have pathological consequences that cause the observed disease trait.

Although all four subjects shared clinical findings, these characteristics were mostly non-specific. Severe feeding difficulties, present from birth, that required intervention (3/4 subjects). The subjects had small size at birth (3/4), with microcephaly (3/4) and severe psychomotor delay, with missed milestones (4/4) at their most recent evaluation. Deep palmar creases (4/4) and slight ulnar deviation of the hands (3/4), combined with a high arched palate (3/4) were also common. No patient had the typical 'BOS posture' of elbow and wrist flexion, or of myopia or trigonocephaly (0/4).

The phenotype present in the three affected individuals varies in both presentation and severity, a phenomenon that is also reported in subjects with *ASXL1 *mutation. Several factors may account for this. First, truncating mutations occurring earlier in the gene seem to be associated with a more severe phenotype, with truncating mutations at the extreme 3' end of the gene yielding no observed phenotype. Interestingly, this does not seem to be the case for *ASXL1 *[2]. Additional subjects will be needed for further genotype-phenotype analysis to address a potential polarity hypothesis [23]. Second, because of the importance of the *ASXL *gene family in very early development, the time at which the mutation arose may also influence the phenotypic outcome; mutations that occurred in the parental gametes could convey a more severe phenotype than those arising post-zygotically or during later embryogenesis [24]. Third, ASXL proteins form complexes with other proteins, and have been shown to influence *Trx *gene mutations in flies [12]. Mutational load and other epistatic effects may contribute to the observed phenotype, and we could not discern such alleles using our *de novo *variant approach. Finally, epigenetic factors may contribute to the phenotype. In mice, homozygous *Asxl2 *mutations can lead to two primary outcomes: around 20% are born very small and die by the age of 2 months, whereas the remaining 80% are smaller at birth but gain weight normally and are successfully weaned [25]. Thus, other factors may contribute to the penetrance and/or expressivity of *de novo ASXL3 *mutations in humans, and severe phenotypes could be atypical.

The condition defined molecularly in the current study is phenotypically distinct from, but with similar and overlapping features to BOS. This is probably the consequence of functional overlap between the causative candidate genes *ASXL1 *and *ASXL3*, which are both developmentally important putative polycomb genes. Differentiating two phenotypically similar syndromes based on clinical presentation alone is challenging, and is further complicated by phenotypic variability. Molecular methods permit an objective means to establish and secure a diagnosis. Moreover, these methods now enable comparative analyses between novel and well-described syndromes to make use of evolutionary genetics in addition to phenotypic features in disease nosology. This allows a distinct molecular diagnosis, and increases diagnostic capabilities for rare syndromes. Interestingly, in this study, the subjects were identified not by establishing phenotypic overlap between them, but rather by identifying that they shared *de novo *nonsense mutations in the identical genes, and that mutations in a related gene, *ASXL1*, resulted in a similar phenotype. In particular, subjects 3 and 4 were identified from a small clinical cohort (*n *= 192) of individuals with psychomotor delay, based upon the presence of rare truncating *ASXL3 *mutations, which were later determined to be *de novo*. This is a novel way in which molecular diagnostics can help foster international and inter-institutional collaborations that will be vital to both solving the multitude of very rare diseases and to functionally annotating the human genome.

## Conclusion

*De novo *truncating *ASXL3 *mutations are very likely the cause of a novel syndrome. The ability to communicate and share genotype findings can elucidate novel disorders that would be challenging to discover based on phenotypic data alone.

## List of abbreviations

BCM-HGSC: Baylor College of Medicine Human Genome Sequencing Center; BOS: Bohring-Opitz Syndrome; BWA: Burrows-Wheeler Aligner; CdLS: Cornelia de Lange Syndrome; miRNA: microRNA; MPIMG: Max Planck Institut für molekulare Genetik; NMD: nonsense-mediated decay; qPCR: quantitative polymerase chain reaction; SNV: single-nucleotide variant.

## Competing interests

The authors declare that they have no competing interests.

## Authors' contributions

Baylor College Medicine Group (subjects 1, 3 and 4): MNB and RAG conceived and planned the experimentsl MNB, JRL, VRS, RAG, and KWG prepared the manuscript; VRS, BHG, KWG, KJ, and JRL enabled clinical characterization and sample collection; DMM and YY conducted high-throughput and validation sequencing; and MNB conducted data analysis and interpretation. Berlin group (subject 2): HHR conceived and planned the experiments and helped prepare the manuscript; TFW enabled clinical characterization and sample collection, and performed database screening; WC conducted high-throughput sequencing; HH conducted data analysis and interpretation; and LM validated the WES and WGS results. All authors read and approved the final manuscript.

## Supplementary Material

Additional file 1 **Figure S1**. T2-weighted brain magnetic resonance images of subject 4. Click here for file

Additional file 2 **Table S1**. Sequencing approach, data alignment, and coverage statistics for all subjects. Click here for file

Additional file 3 **Table S2**. Mutation nd effect of the mutation on protein and the local region around the mutation. Click here for file

Additional file 4 **Figure S2**. Normalized expression of ASXL1 and ASXL3 proteins in adult human tissues. Click here for file

Additional file 5 **Figure S3**. Expression profile of ASXL3 and ASXL1 proteins across three subjects. Click here for file

Additional file 6 **Figure S4**. Sanger chromatogram of gDNA (top) and cDNA (indicated) derived from *ASXL3 *mRNA. Click here for file

Additional file 7 **Figure S5**. Known truncating mutations in *ASXL1*. Click here for file
