# Supplementary material for: De novo truncating mutations in ASXL3 are associated with a novel clinical phenotype with similarities to Bohring-Opitz syndrome
Source: Genome Med. 2013 Feb 5;5(2):11. doi: 10.1186/gm415 (PMC3707024; doi:10.1186/gm415)
Supplement: Additional file 1 — Figure S1. T2-weighted brain magnetic resonance images of subject 4. [file gm415-S1.docx]

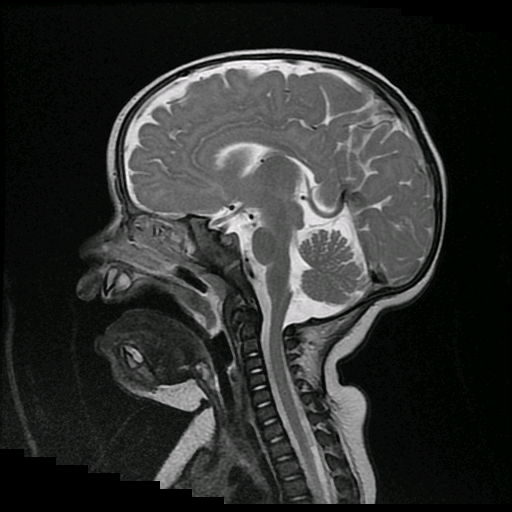

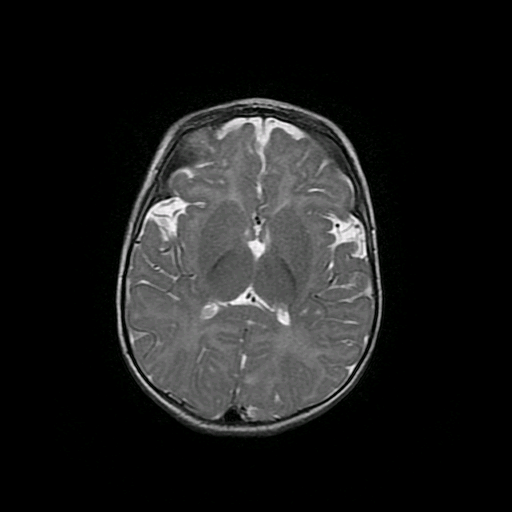

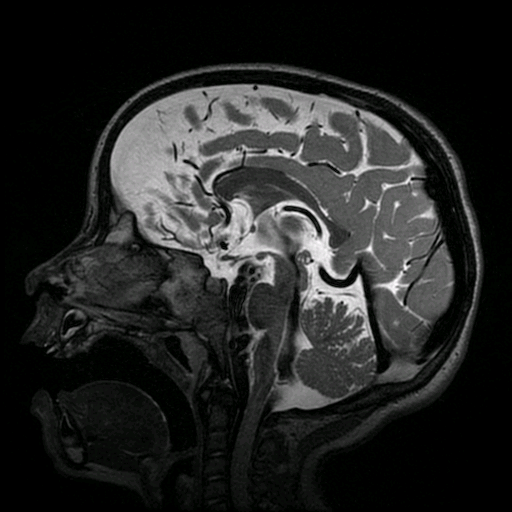

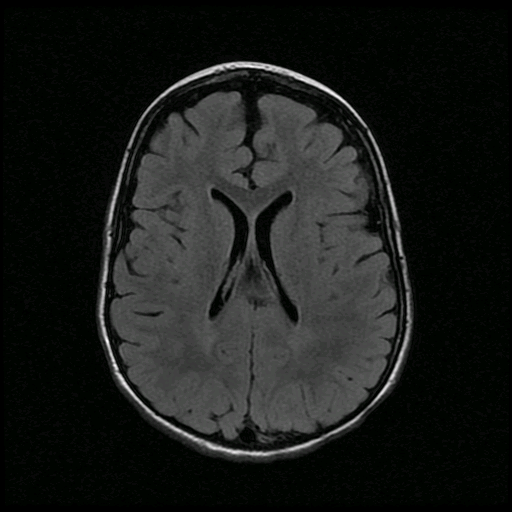


**B**

**D**

**C**

**A**

**Figure S1.** T2-weighted brain MR images of subject 4, at age 4 months (A, sagittal; B axial) and 3 4/12 years (C, sagittal; D axial T1-weighted image). Enlarged extra-axial fluid spaces and mild brain stem hypoplasia become more prominent over time due to global cerebral volume loss. Hypoplasia/dysplasia of bilateral cerebellar tonsils and mild inferior vermian hypoplasia are noted.
