# Supplementary material for: De novo truncating mutations in ASXL3 are associated with a novel clinical phenotype with similarities to Bohring-Opitz syndrome
Source: Genome Med. 2013 Feb 5;5(2):11. doi: 10.1186/gm415 (PMC3707024; doi:10.1186/gm415)
Supplement: Additional file 2 — Table S1. Sequencing approach, data alignment, and coverage statistics for all subjects. [file gm415-S2.docx]

**Table S1.** Sequencing approach, data aligned and coverage statistics for all subjects. Percentage of targeted bases at 20X coverage or higher, shown for exome data.

| **Family** | **Subject** | **Sequence Type** | **Total Sequence Aligned (Gbp)** | **Average Coverage (X-fold)** |
| --- | --- | --- | --- | --- |
| 1 | Father | Exome (Illumina) | 9.3 | 111 (93% @ 20x+) |
| 1 | Mother | Exome (Illumina) | 9.4 | 97 (90% @ 20x+) |
| 1 | Affected | Exome(Illumina) | 8.2 | 90 (89% @ 20x+) |
| 2 | Father | Whole genome (CG) | 150.1 | 51 (95%@20x+) |
| 2 | Mother | Whole genome (CG) | 154.0 | 53 (95%@20x+) |
| 2 | Unaffected sibling | Whole genome (CG) | 151.5 | 52 (94%@20x+) |
| 2 | Affected | Whole genome (CG) | 150.7 | 52 (95%@20x+) |
| 2 | Father | Exome (Illumina) | 9.2 | 127 (93%@20x+) |
| 2 | Mother | Exome (Illumina) | 10.2 | 134 (94%@20x+) |
| 2 | Unaffected sibling | Exome (Illumina) | 10.4 | 132 (94%@20x+) |
| 2 | Affected | Exome (Illumina) | 8.4 | 116 (93%@20x+) |
| 3 | Father | Sanger only | n/a | n/a |
| 3 | Mother | Sanger only | n/a | n/a |
| 3 | Affected | Exome (Illumina) | 14.7 | 199 (97.6% @20x+) |
| 4 | Father | Sanger only | n/a | n/a |
| 4 | Mother | Sanger only | n/a | n/a |
| 4 | Affected | Exome (Illumina) | 14.6 | 201 (97.6%@20x+) |
