# Supplementary material for: De novo truncating mutations in ASXL3 are associated with a novel clinical phenotype with similarities to Bohring-Opitz syndrome
Source: Genome Med. 2013 Feb 5;5(2):11. doi: 10.1186/gm415 (PMC3707024; doi:10.1186/gm415)
Supplement: Additional file 3 — Table S2. Mutation nd effect of the mutation on protein and the local region around the mutation. [file gm415-S3.docx]

**Table S2.** Discovered mutation, mutation effect on protein and local region around mutation.

| **Subject** | **Chromosome** | **Mutation** | **Effect** | **Local Region** |
| --- | --- | --- | --- | --- |
| 1 | Chr18 | g.31318578C>T | p.Q404X | ACAA[C/T]AGCC |
| 2 | Chr18 | g.31318764C>T | p.Q466X | ATGC[C/T]AGGA |
| 3 | Chr18 | g.31319343_31319346delACAG | p.T659fsX41 | AAAT[ACAG/-]ACAA |
| 4 | Chr18 | g. 31318789_insT | p.P474fsX0 | AATACC[-/T]TGAAT |
