# Supplementary material for: De novo truncating mutations in ASXL3 are associated with a novel clinical phenotype with similarities to Bohring-Opitz syndrome
Source: Genome Med. 2013 Feb 5;5(2):11. doi: 10.1186/gm415 (PMC3707024; doi:10.1186/gm415)
Supplement: Additional file 4 — Figure S2. Normalized expression of ASXL1 and ASXL3 proteins in adult human tissues. [file gm415-S4.docx]

**Figure S2**. Normalized expression of ASXL1 and ASXL3 in adult human tissues. Adapted from GeneCards^1^.
