# Supplementary material for: De novo truncating mutations in ASXL3 are associated with a novel clinical phenotype with similarities to Bohring-Opitz syndrome
Source: Genome Med. 2013 Feb 5;5(2):11. doi: 10.1186/gm415 (PMC3707024; doi:10.1186/gm415)
Supplement: Additional file 5 — Figure S3. Expression profile of ASXL3 and ASXL1 proteins across three subjects. [file gm415-S5.docx]

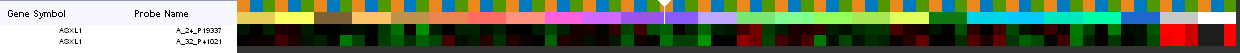

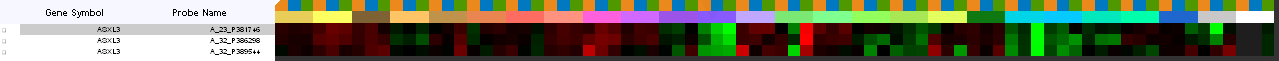


Insula

Cingulate gyus

Claustrum

Amygdala

White matter

Sulsi and spaces

ASXL3

ASXL1

**Figure S3.** Expression profile of ASXL3 (3 probes, indicated) and ASXL1 (2 probes, indicated), across 3 subjects (top row, orange, blue, green), and multiple structures of the brain (second row, colored) to show differences in expression (indicated). Adapted from Allen Brain Atlas^2^.
