# Supplementary material for: De novo truncating mutations in ASXL3 are associated with a novel clinical phenotype with similarities to Bohring-Opitz syndrome
Source: Genome Med. 2013 Feb 5;5(2):11. doi: 10.1186/gm415 (PMC3707024; doi:10.1186/gm415)
Supplement: Additional file 6 — Figure S4. Sanger chromatogram of gDNA (top) and cDNA (indicated) derived from ASXL3 mRNA. [file gm415-S6.docx]

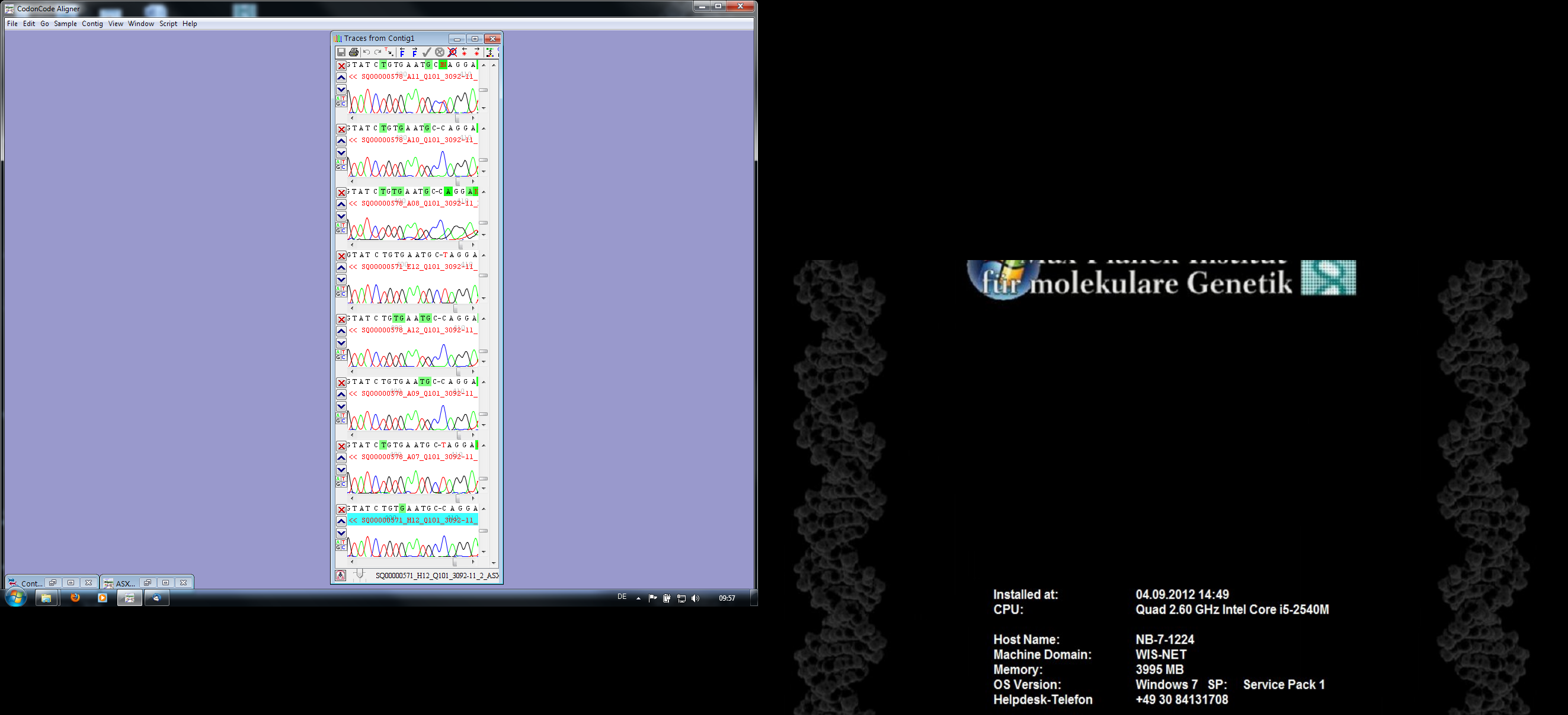

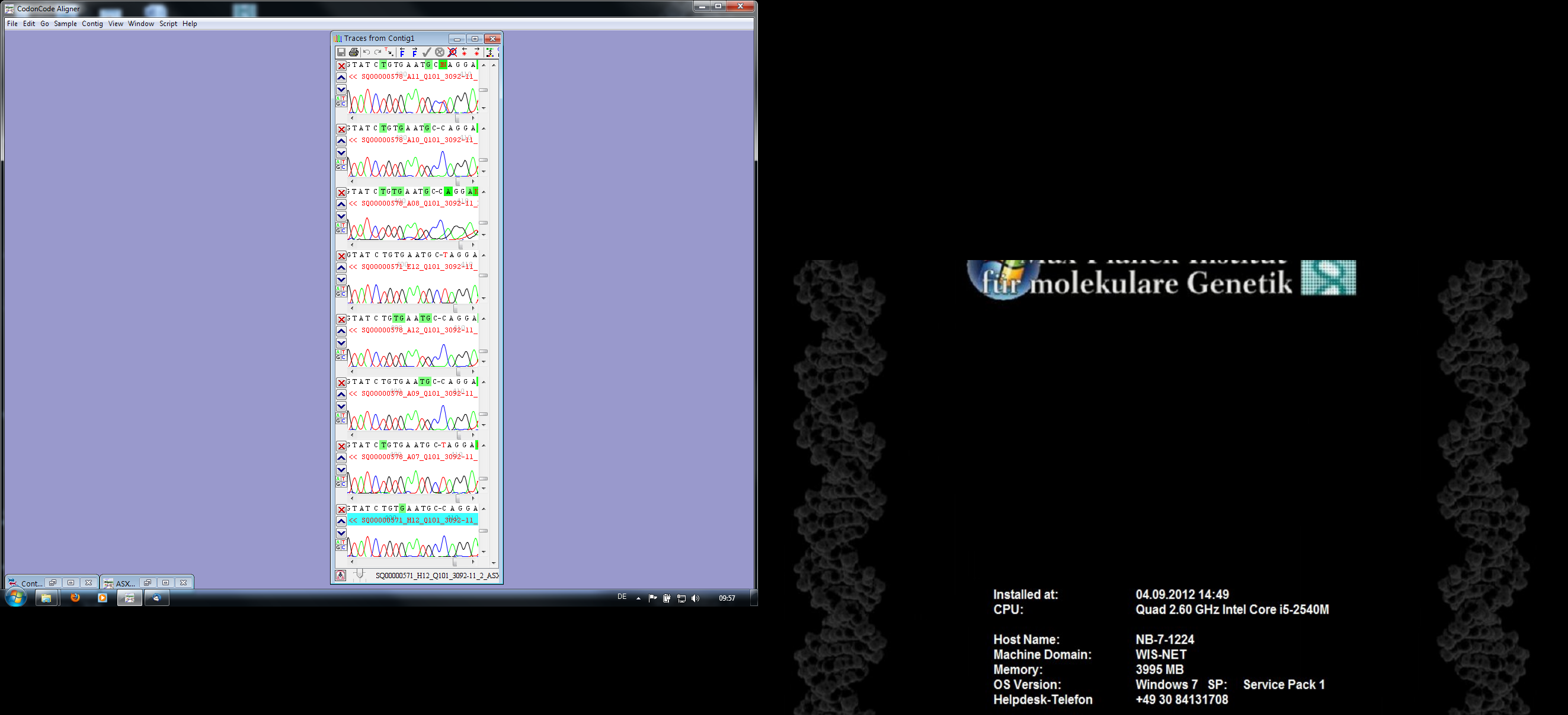

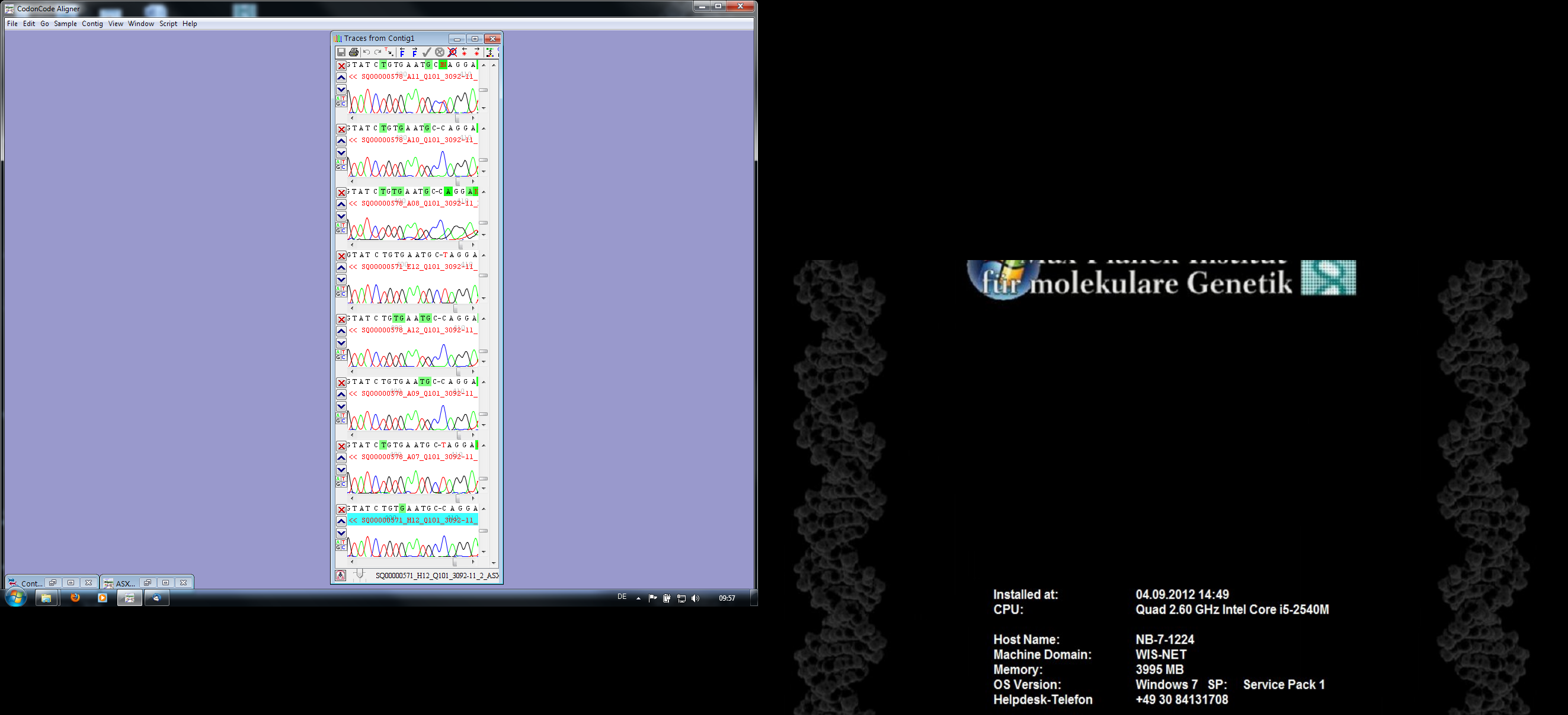

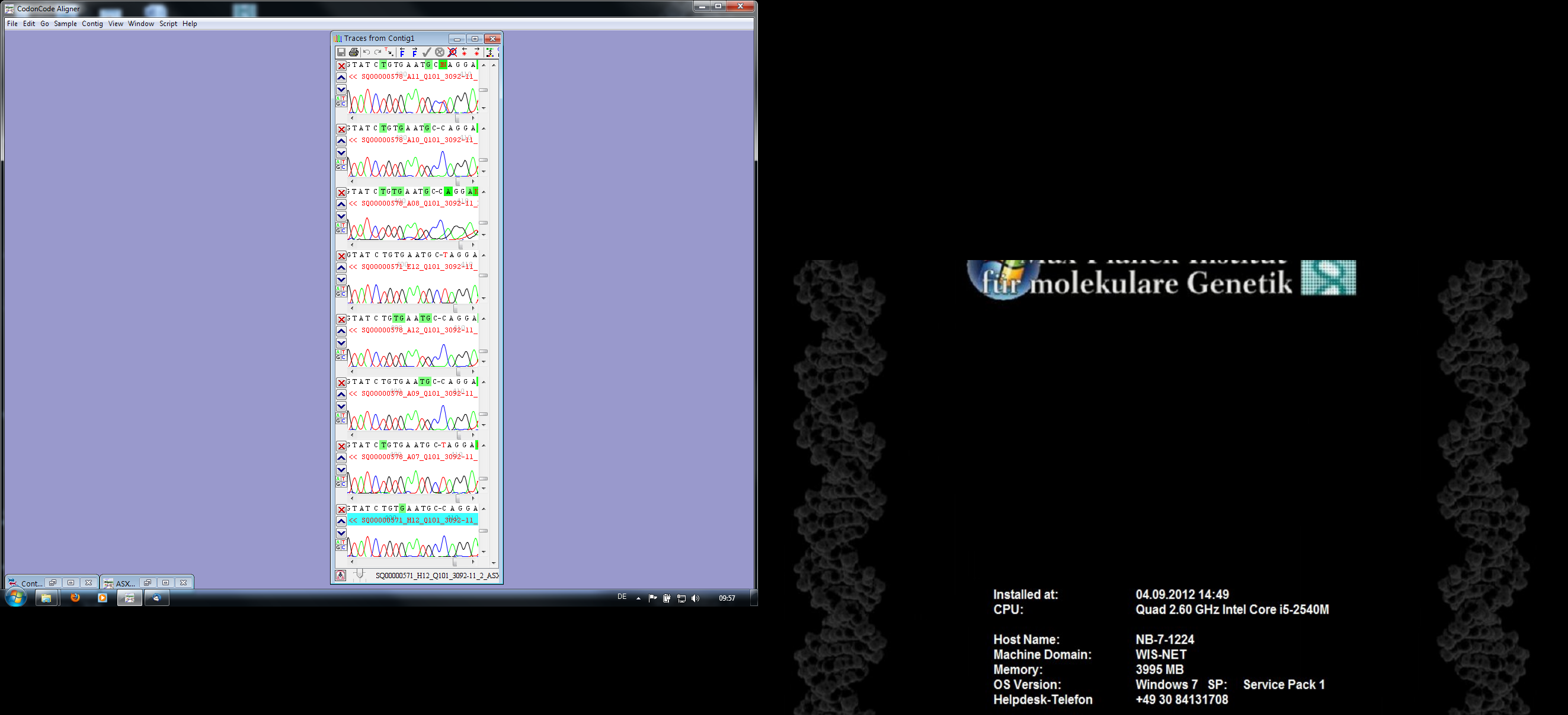

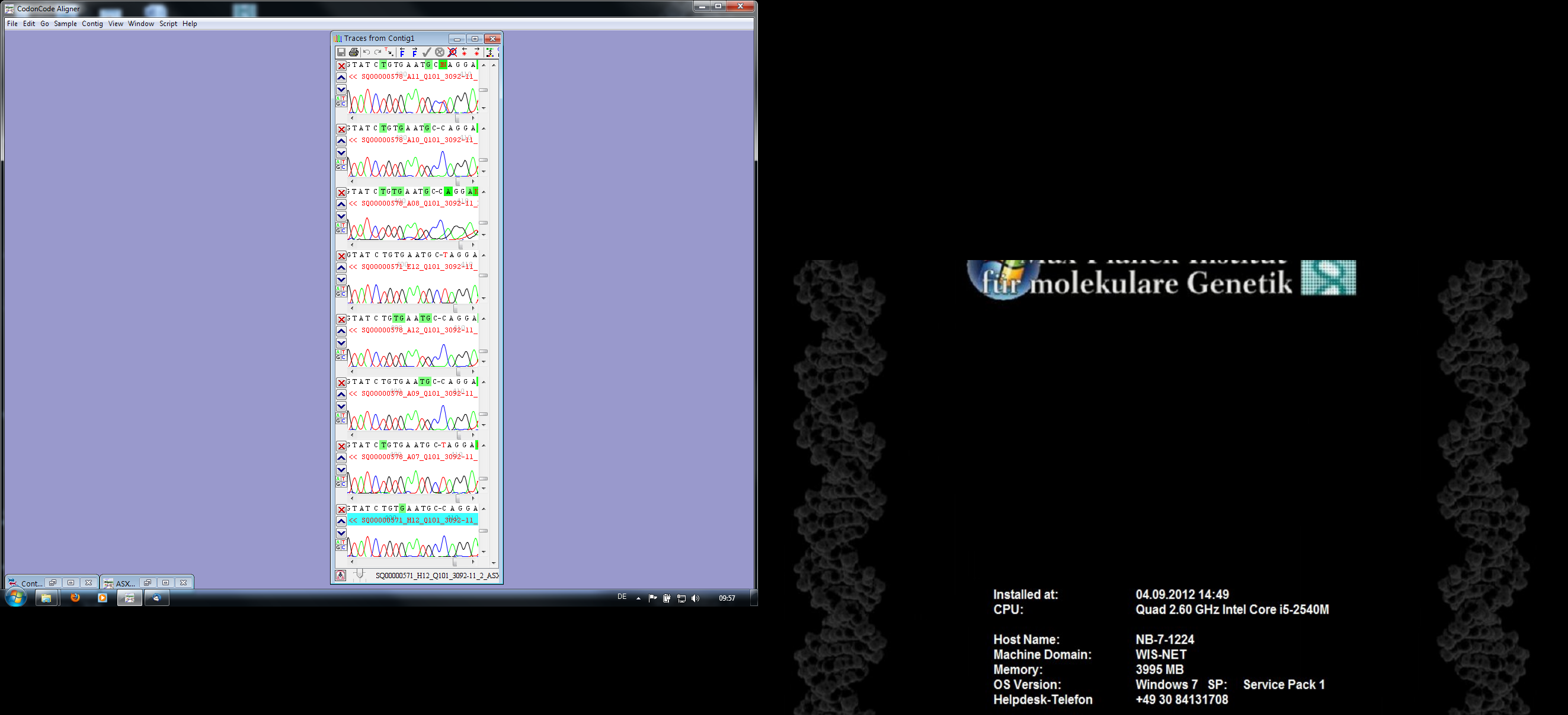

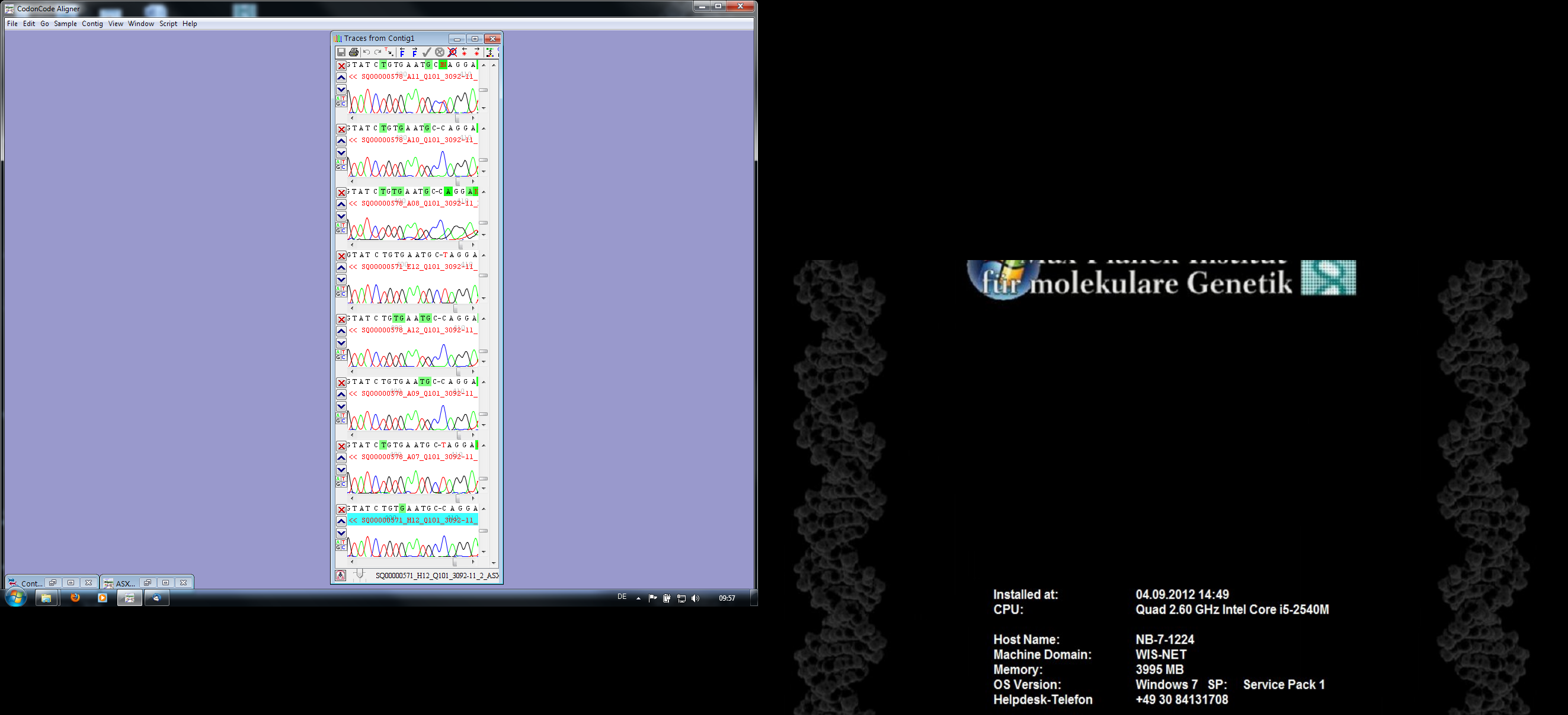

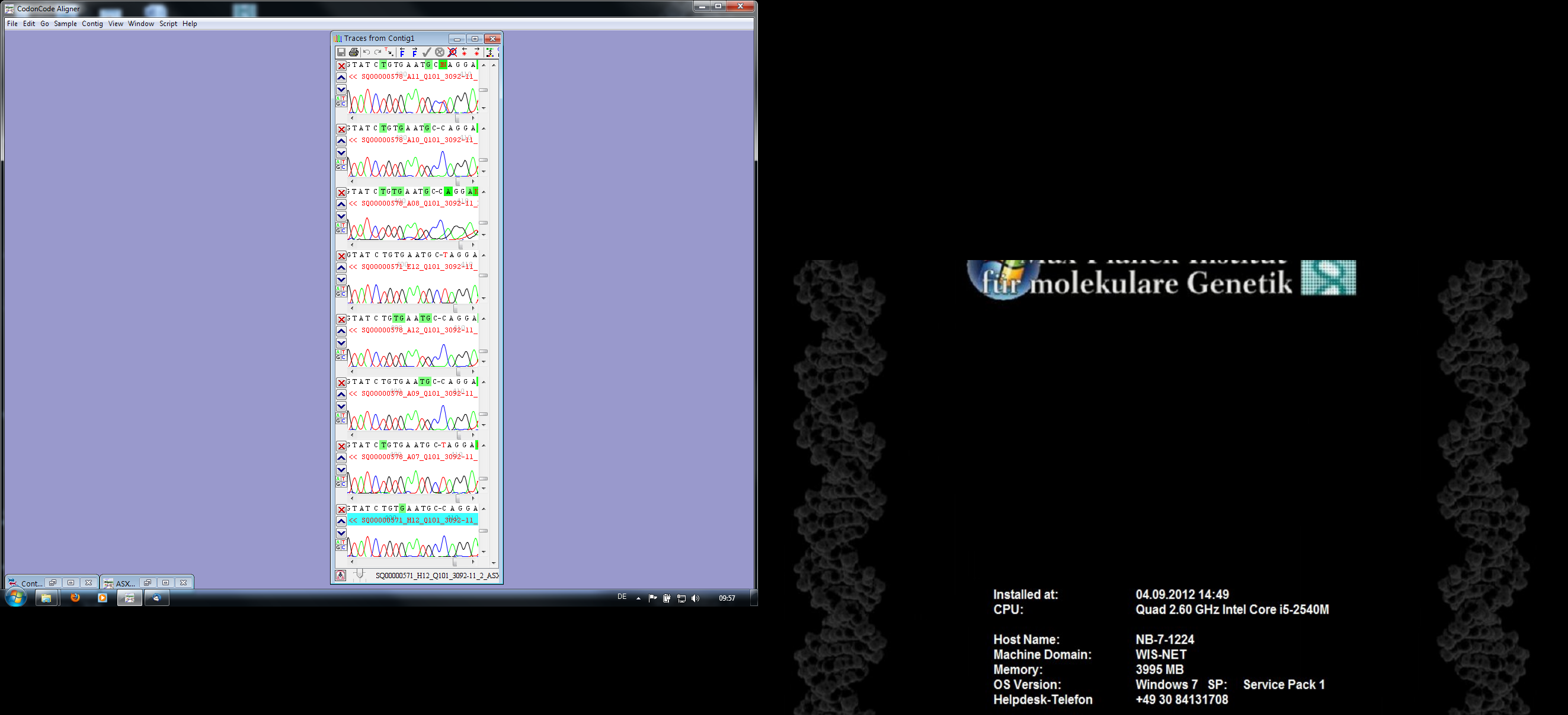

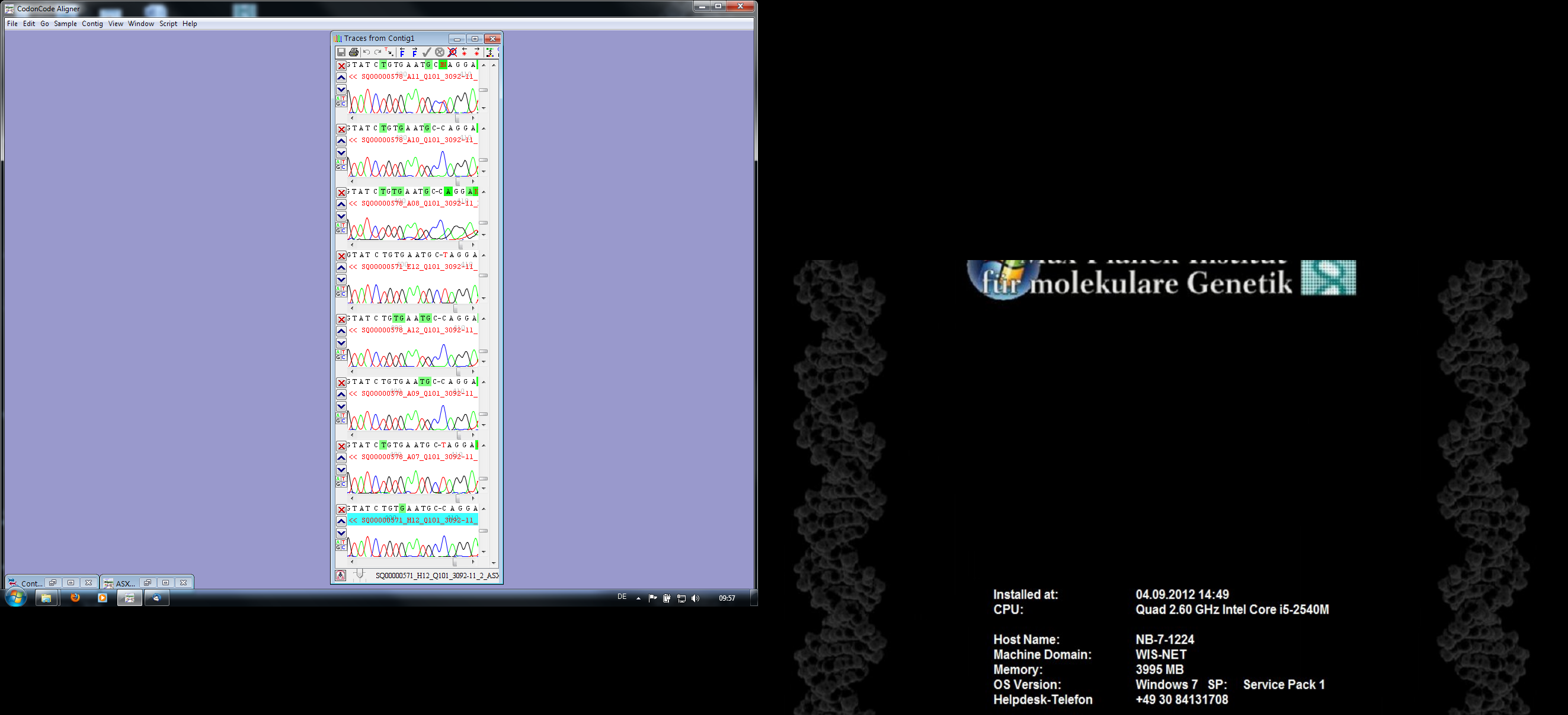


C/T

Subject 2, gDNA

Subject 2, cDNA #1

Subject 2, cDNA #2

Subject 2, cDNA #3

Subject 2, cDNA #4

Subject 2, cDNA #5

Subject 2, cDNA #6

Subject 2, cDNA #7

**Figure S4.** Sanger chromatogram of gDNA (top) and cDNA (indicated) derived from *ASXL3* mRNA fragment spanning exons 10 and 11 amplified from subject 2. gDNA shows presence of both wild-type allele, cytosine (red) and mutant allele, thymine (blue). Multipel cDNA preparations showed only one or the other allele.
