# Supplementary material for: De novo truncating mutations in ASXL3 are associated with a novel clinical phenotype with similarities to Bohring-Opitz syndrome
Source: Genome Med. 2013 Feb 5;5(2):11. doi: 10.1186/gm415 (PMC3707024; doi:10.1186/gm415)
Supplement: Additional file 7 — Figure S5. Known truncating mutations in ASXL1. [file gm415-S7.docx]

200

1541

400

600

800

1000

1200

ASXL2

ASXL3

**Figure S5.** Known truncating mutations in ASXL1. Disease causing mutations are shown in red (Hoischen et al) and orange (Magrini et al). Benign truncating mutations are shown in green (dbSNP) and purple (ESP5400). Amino acid similarity is shown between ASXL1 and ASXL2 and ASXL3 (highest similarity to lowest: red, pink, green, blue).
